# Levonorgestrel-releasing intrauterine system vs. systemic medication or blank control for women with dysmenorrhea: Systematic review and meta-analysis of randomized controlled trials

**DOI:** 10.3389/fgwh.2022.1013921

**Published:** 2022-11-02

**Authors:** Jing Wang, Ke Deng, Ling Li, Yi Dai, Xin Sun

**Affiliations:** ^1^ Chinese Evidence-based Medicine Center, West China Hospital, Sichuan University, Chengdu, China; ^2^ Department of Obstertrics and Gynecology, Peking Union College Hospital, Chinese Academy of Medical Sciences & Peking Union Medical College, National Clinical Research Center for Obstetrics & Gynecologic Diseases, Beijing, China

**Keywords:** dysmenorrhea, efficacy, levonorgestrel-releasing intrauterine system, meta-analysis, safety

## Abstract

**Aims:**

To compare efficacy and safety of the levonorgestrel-releasing intrauterine system (LNG-IUS) with systemic medication or blank control in the treatment of dysmenorrhea.

**Methods:**

PubMed, EMBASE, the China National Knowledge Infrastructure (CNKI) and Wanfang Data were searched to collect randomized controlled trials (RCTs) comparing LNG-IUS with systemic medication or blank control among women diagnosed with primary dysmenorrhea or secondary dysmenorrhea (adenomyosis or endometriosis) from inception to 2020.04. Der Simonian-Laird random-effect model was used to pool data.

**Results:**

Seventy-one RCTs (6551 patients) were included. Overall bias risk was medium. Sixty-two articles enrolled patients with adenomyosis; LNG-IUS significantly reduced the visual analogue scale (VAS) score compared with the systemic medication group among adenomyosis women at 3 months (standardized mean difference (SMD) = −0.81, 95% confidence interval (CI) −1.22 to −0.40); 6 months (SMD = −1.25, 95%CI: −1.58 to −0.92); 9 months (SMD = −1.23, 95%CI: −1.63 to −0.83); 12 months (SMD = −1.66, 95%CI: −2.14 to −1.18). No difference was found in the incidence of irregular vaginal bleeding (16 RCTs; RR = 0.91, 95%CI: 0.62−1.33, *P* = 0.63, *I*^2 ^= 4%) and other adverse outcomes. Sensitivity analysis regarding randomization methods was robust. Nine RCTs enrolled endometriosis women. Pooling results showed no significant difference between LNG-IUS and systemic medication treatment in terms of VAS at 6 months (SMD = −0.27, 95% CI: −0.97–0.43). Moreover, LNG-IUS was associated with higher risk of irregular vaginal bleeding (26.8% vs. 0).

**Conclusions:**

LNG-IUS was associated with a reduced severity of dysmenorrhea compared with systemic medication; it was also beneficial for better control of menstrual blood loss and fewer adverse outcomes. Owing to small sample sizes, further well-designed RCTs are warranted to confirm these findings and long-term effects of LNG-IUS in the treatment of dysmenorrhea.

**Systematic Review Registration:**

https://www.crd.york.ac.uk/prospero/, identifier: CRD42021228343.

## Introduction

Dysmenorrhea is the most common gynecologic disorder, and it affects about 60%–90% women of reproductive age (1, 2). It is characterized by crampy lower abdominal pain, nausea, vomiting, and headaches, and it has a significant impact on ability to study, ability to work, and daily life (3). Oral contraceptive pills (OCPs) are used as first-line hormonal therapy for dysmenorrhea. They reduce uterine production of prostaglandin, which causes experienced pain (3). However, owing to the strict dosing cycle required when taking the pills, there is a risk of forgetting to take them.

The levonorgestrel-releasing intrauterine system (LNG-IUS) is an IUD. And LNG-IUS has three models, respectively are Mirena, Kyleena, Jaydess. But only Mirena went into public in China. Mirena releases 20 μg of levonorgestrel per day into the uterine cavity for a period of 5 years. Kyleena contains 19.5 mg of levonorgestrel (LNG) released *in vivo* at a rate of approximately 17.5 mcg/day after 24 days. This rate decreases progressively to 9.8 mcg/day after 1 year and to 7.4 mcg/day after 5 years. Levonorgestrel belongs to the class of medications called progestins, which is a hormone produced by the ovaries. This is a soft, flexible T-shaped contraceptive (birth control) device that is placed inside the uterus (intrauterine device or IUD). The medication is continuously released over a period of 3 years to prevent pregnancy. LNG-IUS was first designed as a contraceptive in 1970; it reduces the experience of pain and avoids the need to regularly take long-term medication. In recent years, LNG-IUS has also been officially approved for the treatment of heavy menstrual bleeding (4) and also been recommended for the treatment of dysmenorrhea when the patients are not currently planning pregnancy (2, 5, 6). But in China, it has not been recommended to women with dysmenorrhea yet. Although several small-sample randomized controlled trials (RCTs) indicate that LNG-IUS is likely beneficial for reducing the pain in women with endometriosis or adenomyosis (7–10), the results were not consistent. Furthermore, there is no existing systematic review about the efficacy and safety of LNG-IUS comparing systemic medication in the treatment of dysmenorrhea. Therefore, we conducted a systematic review and meta-analysis of RCTs to determine the efficacy and safety of LNG-IUS compared to other systemic medication or blank control in women with dysmenorrhea. Additionally, we identify the possible explanations for the heterogenicity through subgroup analysis.

## Methods

### Design

We performed a systematic review with meta-analysis using the protocol registered with PROSPERO (CRD42020203343).

### Eligibility criteria

We included RCTs comparing LNG-IUS with systemic medication or blank control among women diagnosed with primary dysmenorrhea or secondary dysmenorrhea (adenomyosis or endometriosis). We excluded studies that did not report on our interested outcomes and those that were not published in English or Chinese. The primary outcome was defined as the degree of dysmenorrhea (measured using visual analogue scale [VAS] scores) at 3, 6, 9, and 12 months, and blood loss (measured using a pictorial blood loss assessment chart [PBAC] scores, or menstrual blood volume) at 3, 6, 9, and 12 months. The secondary outcome was defined as quality of life (measured using standard scales like short form-questionnaire-36 [SF-36]) and adverse events (irregular vaginal bleeding, amenorrhea, headache, nausea, pelvic pain, acne, ovarian cyst, or weight gain).

### Literature search

We searched PubMed, EMBASE, the China National Knowledge Infrastructure (CNKI) and Wanfang Data, from inception to April 2020, with language limitation to English and Chinese. We used MeSH/Emtree terms and free-text to generate the search strategy, which comprised the terms “levonorgestrel releasing intrauterine system”, “LNG-IUS”, “dysmenorrhea”, “painful menses”, “menstrual pain”, “chronic pelvic pain”, and “dyspareunia”. The details of our search strategy are listed in Supplementary S1.

### Study process

Two paired reviewers who were well-trained in research methodology, independently screened titles, abstracts, and full texts for eligibility; assessed risk of bias; and collected data from eligible studies, using standardized, pilot tested forms with detailed instructions. Reviewers resolved disagreement through discussion or through adjudication by a third reviewer (LL) if agreement could not be reached.

### Risk of bias assessment

We used the Cochrane Handbook 5.1.0 bias assessment tool (11, 12) to assess the risk of bias of included studies. The items include sequence generation, allocation concealment, blinding of participants, blinding of intervention providers, blinding of outcome assessment, incomplete outcome data, selective outcome reporting, and other source of bias. We assigned items that were addressed “definitely or probably yes” to “high risk of bias”; and items that were addressed “definitely or probably no” to “low risk of bias”; items that did not report on our interested information as “unclear”.

### Data extraction

The following information from all eligible studies were collected:

• Study characteristics: first author, country, publication year, study design, inclusion and exclusion criteria, number of study sites, sample sizes, length of follow-up.
• Patient characteristics: age, parity
• Intervention: details of LNG-IUS and systemic medication of the control group
• Outcomes: degree of dysmenorrhea (VAS score), blood loss volume (PBAC score), quality of life, side effects (number of events and patients included for analyses in each group).

### Data analysis

For dichotomous outcomes, we calculated risk ratio (RR) and their 95% confidence intervals (CIs). For continuous outcomes, we calculated mean difference (MD) or standardized mean difference (SMD) and their 95% CIs. Statistical heterogeneity was examined by *I*^2^ and *χ*^2^ test. If *I*^2 ^> 50% or *χ*^2^ test indicated *P* < 0.1, we defined it as statistical significance and we used the DerSimonian-Laird random-effect model to pool data. Subgroup analysis was undertaken to explore the potential heterogeneity based on systemic medication types (mifepristone, gestrinone, methyltestosterone, triptorelin, Desogestrel-Ethinyl Estradiol, drospirosterone-ethinylestradiol, and other types), status of surgery and patient type (primary dysmenorrhea patients vs. secondary dysmenorrhea patients). Sensitivity analysis was also conducted by excluding studies with improper randomization sequence generation or unspecified randomization methods and excluding studies with sample size less than 50. Data analyses were undertaken by Review Manager 5.3.5. Publication bias was examined by Egger’s test and funnel plots.

## Results

**Figure 1:**
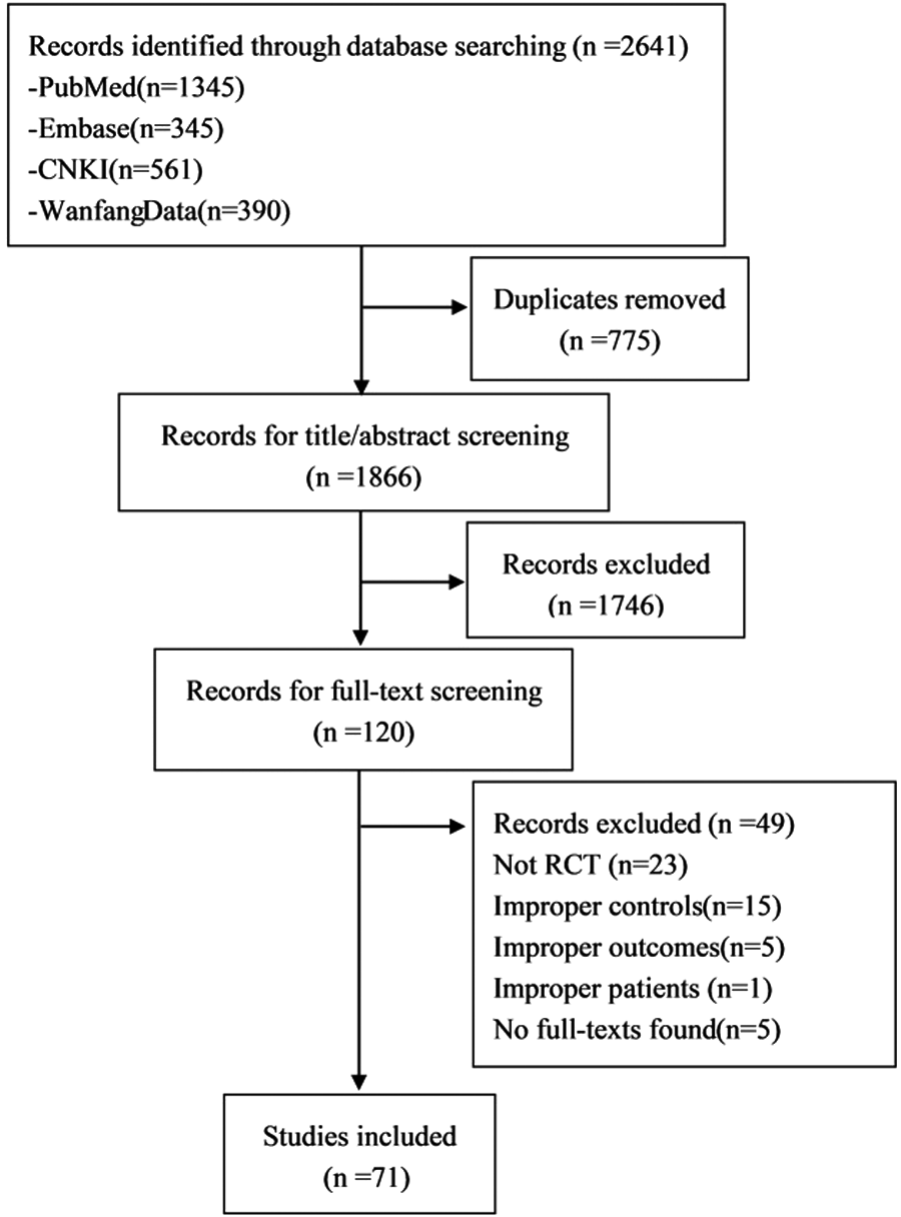
Flow diagram of search result (April 2020).

Among 2641 identified publications, reviewers selected 71 RCTs (13–76, 78–84), enrolling 6551 patients, to be included in this meta-analysis (Figure 1).

### Study characteristics

Of 71 included studies, eight (73–76, 78, 82–84) were published in English and 63 (13–72, 79–81, 86) were published in Chinese. The included studies were conducted in China mainland (*n* = 64), Brazil (*n* = 3), Egypt (*n* = 1), Thailand (*n* = 1), Turkey (*n* = 1), Taiwan (*n* = 1). Seventy studies were single center clinical trials, and one study (74) was a multicenter clinical trial. All the LNG-IUS used in article are Mirena. The details of the characteristics of the study are summarized in Supplementary Table S1.

### Risk of bias assessment

The results of risk of bias assessment are presented in Supplementary Table S2. Twenty-nine studies described adequate randomization methods while the remaining 42 studies did not report randomization methods. Eight studies reported using sealed and opaque envelopes for allocation concealment. One study reported methods for blinding participants and another trial reported methods for blinding outcome assessment. Six studies reported loss of follow-up. The overall risk of bias was medium. Sensitivity analysis considering whether randomization methods were adequate did not show a significant difference (See the online Supplementary Table S2).

### Efficacy and safety results in women with adenomyosis

**Figure 2:**
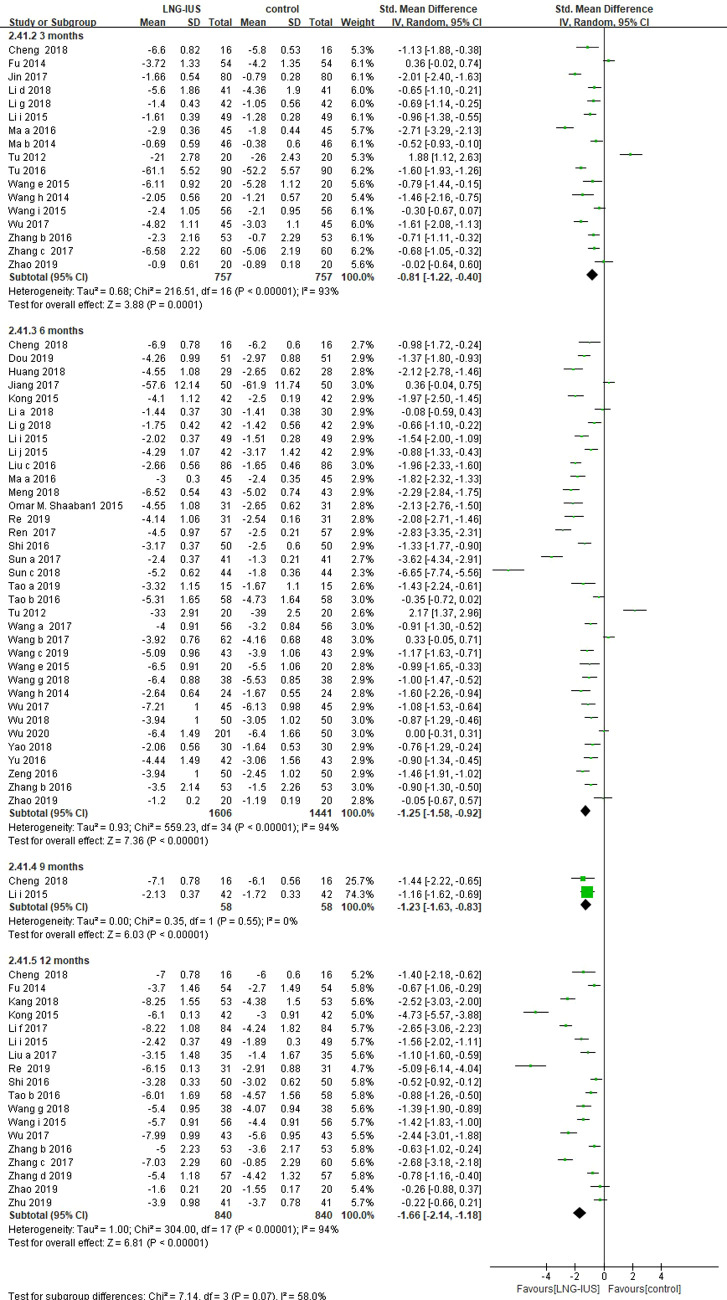
VAS score among adenomyosis patients receiving treatment with LNG-IUS vs. systemic medication in randomized controlled studies.

Of the 71 RCTs included, 62 (13–23, 25–36, 38–49, 51–56, 58–72, 76, 78–81, 86) enrolled women patients with adenomyosis, among which 42 (13, 16, 17, 19–23, 25, 27, 31, 32, 34–36, 38–42, 44, 45, 48, 49, 51–53, 55, 56, 58, 60–62, 64, 66, 68–71, 76, 81, 86) studies reported VAS score. Pooling data from these 42 RCTs showed that LNG-IUS significantly reduced the VAS score in the comparison of a medication group among women with adenomyosis at 3 months (17 RCTs; SMD = −0.81, 95% CI = −1.22–(−0.4), *P* < 0.0001; *I*^2 ^= 93%); 6 months (36 RCTs; SMD = −1.25, 95% CI: −1.58–(−0.92), *P* < 0.0001; *I*^2 ^= 94%); 9 months (2 RCTs; SMD = −1.23, 95% CI: −1.63–(−0.83), *P* < 0.0001, *I*^ ^= 0); and 12 months (18 RCTs; SMD = −1.66, 95% CI: −2.14–(−1.18), *P* < 0.0001, *I*^2 ^= 94%; Figure 2).

Pooling across 16 (16, 17, 27, 32, 36, 49, 53, 55, 56, 60, 62, 65, 70, 71, 78) trials reporting PBAC score showed that the reduction of PBAC score was greater in LNG-IUS groups at 3 months (6 RCTs; MD = −7.33, 95% CI: −11.39–(−3.27), *P* = 0.0004, *I*^2 ^= 36%) and 6 months (15 RCTs; MD = −12.41, 95% CI: −16.18–(−8.65), *P* < 0.0001, *I*^2 ^= 96%). However, no difference was identified between these groups at 9 months (2 RCTs; MD = −2.78, 95% CI: −6.89–1.34, *P* = 0.19, *I*^2 ^= 0%) and 12 months (3 RCTs; MD = −12.13, 95% CI: −25.84–1.58, *P* = 0.08, *I*^2 ^= 94%; Supplementary Figure S1).

Nineteen RCTs (21, 33–36, 38, 41, 42, 44, 45, 48, 52, 54, 63, 64, 66, 69, 86) that reported menstrual blood loss were included for analysis. The pooling results showed that the reduction in menstrual blood loss was greater in the LNG-IUS group at 3 months (2 RCTs; MD = −29.52, 95% CI: −74.35–15.31), *P* < 0.0001, *I*^2 ^= 98%), 6 months (15 RCTs; MD = −18.97, 95% CI: −28.27–(−9.67), *P* < 0.0001, *I*^2 ^= 96%), and 12 months (7 RCTs; MD = −40.27, 95% CI: −53.64–(−26.90), *P* < 0.0001, *I*^2 ^= 96%; Supplementary Figure S2).

As for quality-of-life (QoL) assessment, Feng (18) reported quality of life using the Nottingham Health Profile (NHP, wherein high scores indicated good outcomes) including pain, vigor, sleep, emotion, activity ability, and social loneliness. The results showed no difference in the changes in NHP scores at 6 months from baseline between the LNG-IUS and gestrinone group (*P* > 0.05). Li (28) reported quality of life using the Short-Form 36 Health Survey Questionnaire (SF-36; wherein high scores indicated good outcomes). The results showed that LNG-IUS improved SF-36 score compared to triptorelin in both physical and psychological areas after 6 months follow-up (*P* < 0.001).

**Figure 3:**
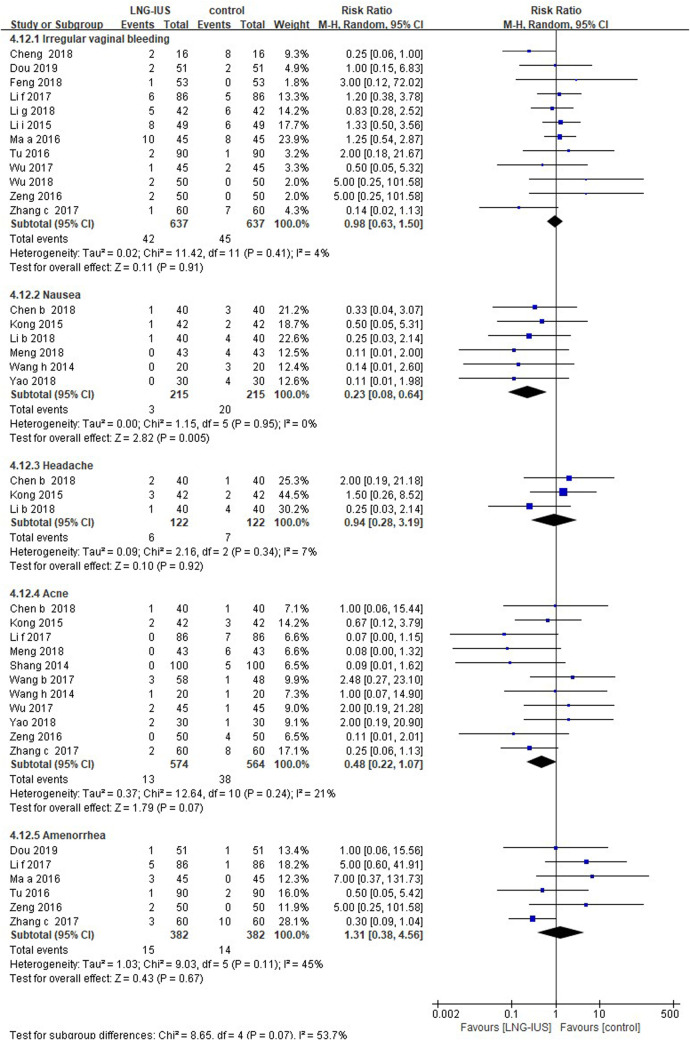
Adverse events among adenomyosis patients receiving treatments of LNG-IUS vs. systemic medication in randomized controlled studies.

There was no significant difference in the incidence of irregular vaginal bleeding (16 RCTs; RR = 0.91, 95% CI: 0.62–1.33, *P* = 0.63, *I*^2 ^= 4%), headache (3 RCTs; RR = 0.94, 95% CI: 0.28–3.19, *P* = 0.92, *I*^2 ^= 7%), acne (11 RCTs; RR = 0.48, 95% CI: 0.22–1.07, *P* = 0.07, *I*^2 ^= 21%), or amenorrhea (7 RCTs; RR = 1.06, 95% CI: 0.34–3.3, *P* = 0.92, *I*^2 ^= 39%) between two groups. The systemic medication group was associated with a higher risk of nausea (6 RCTs; RR = 0.23, 95% CI: 0.08–0.64, *P* = 0.005, *I*^2 ^= 0%) and menorrhagia (1 RCT; RR = 0.55, 95% CI: 0.30–0.98, *P* = 0.04; Figure 3).

Subgroup analysis by type of systemic medications showed a significant difference in VAS score (*P* < 0.0001), while no significant difference was found in subgroup analysis according to whether the patients received surgery or not (*P* = 0.91; Supplementary Table S4). Triptorelin could better reduce VAS score compared with LNG-IUS (SMD = 2.17, 95% CI:1.37–2.96, *P* < 0.0001) after 6 months of treatment.

Sensitivity analysis showed that results were robust after excluding articles with unspecific randomization (*P* < 0.0001 vs. *P* < 0.001; Supplementary Table S3) and excluding studies with sample size is less than 50(*P* < 0.0001 vs. *P* < 0.0001; Supplementary Table S3). No publication bias was detected using funnel plot and Egger’s test with regard to VAS score at 3, 6, and 12 months, PBAC score at 6 months, menstrual blood loss at 6 months, acne, and irregular vaginal bleeding (*P* > 0.05; Supplementary Table S5).

### Efficacy and safety results in women with endometriosis

**Figure 4:**
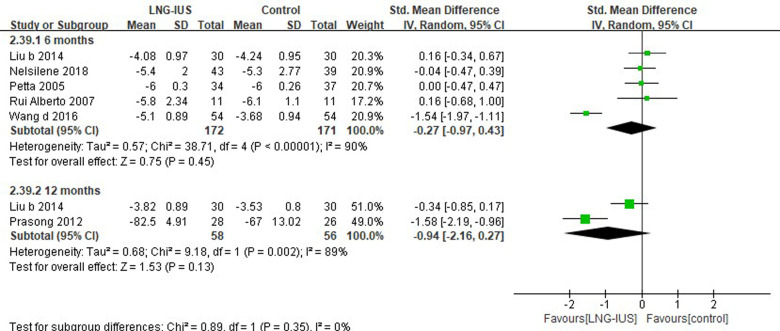
VAS score among endometriosis patients receiving treatments of LNG-IUS vs. systemic medication in randomized controlled studies.

Nine out of all 71 RCTs (37, 50, 57, 73, 74, 82–84, 87) included had enrolled women with (surgically diagnosed) endometriosis, among which six (37, 57, 74, 75, 83, 84) studies reported VAS score. Pooling results showed no significant difference between LNG-IUS and systemic medication treatment in terms of VAS at 6 months (5 RCTs; SMD = −0.27, 95% CI: −0.97–0.43, *P* = 0.45, *I*^2 ^= 90%) and 12 months (2 RCTs; SMD = −0.94, 95% CI: −2.16–0.27, *P* = 0.13, *I*^2 ^= 89%; Figure 4).

As for QoL assessment, Petta (74) used the Psychological General Well-being Index (PGWBI) for the assessment, and no significant difference was identified in the changes in the PGWBI scores from baseline between the LNG-IUS and Triptorelin groups (*P* = 0.78). Nelsilene (83) reported on QoL using Endometriosis Health Profile-30 (EHP-30), and their results showed that EHP-30 was significantly higher in a LNG-IUS group in emotional situation (*P* = 0.04) and self-image (*P* = 0.04) when compared to an etonogestrel implant group. Prasong (75) used the Short-Form 36 Health Survey Questionnaire (SF-36) to assess patients’ QoL. The results showed that the SF-36 at 12 months was higher in the LNG-IUS group than in the expectant group in physical domains (*P* < 0.05) but there was no difference in psychological domains (*P* = 0.229).

**Figure 5:**
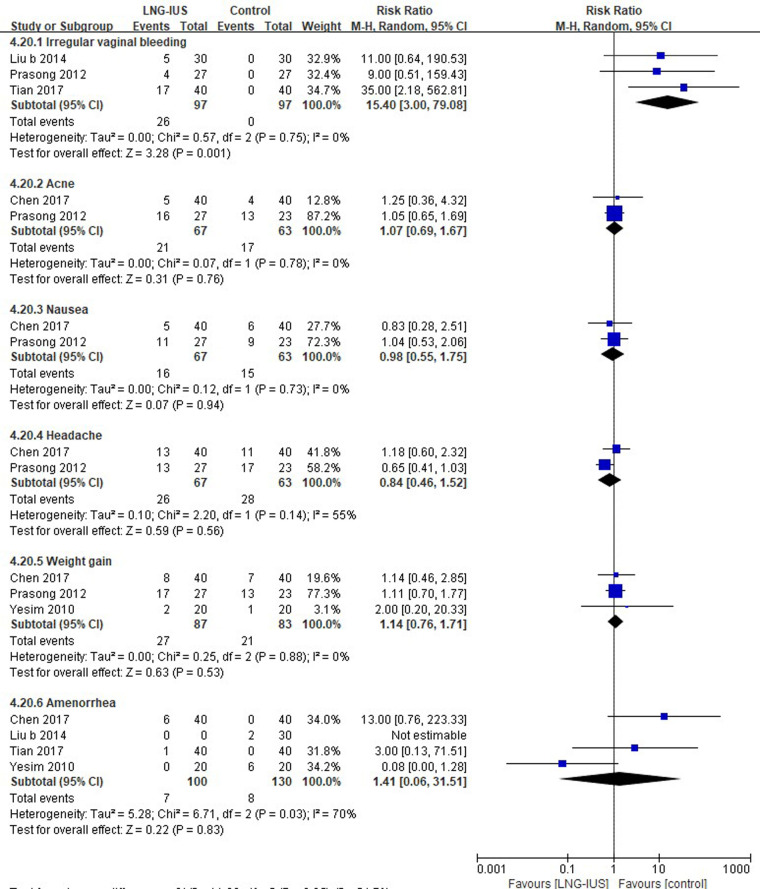
Adverse events among endometriosis patients receiving LNG-IUS vs. systemic medication.

Three studies (37, 50, 75) reporting irregular vaginal bleeding were included. LNG-IUS was associated with a higher risk of irregular vaginal bleeding (26.8%), and no similar events were observed in the control group. The results demonstrated that no significant differences were found for acne (2 RCTs; RR = 1.07, 95% CI: 0.69–1.67, *P* = 0.07, *I*^2 ^= 0%), nausea (2 RCTs; RR = 0.98, 95% CI: 0.55–1.75, *P* = 0.94, *I*^2 ^= 0%), headache (2 RCTs; RR = 0.84, 95% CI: 0.46–1.52, *P* = 0.56, *I*^2 ^= 55%), weight gain (3 RCTs; RR = 1.14, 95% CI: 0.76–1.71, *P* = 0.53, *I*^2 ^= 0%), or amenorrhea (4 RCTs; RR = 1.41, 95% CI: 0.06–31.51, *P* = 0.83, *I*^2 ^= 70%) between the two groups (Figure 5).

Owing to the small sample size of included studies, we did not perform subgroup analysis by systemic medication type. No significance was found in subgroup analysis according to surgical status, which examined whether surgery previously undergone by patients influenced the efficacy of LNG-IUS. (Supplementary Table S4).

Sensitivity analysis showed that results were robust after excluding articles with unspecific randomization (*P* = 0.45 vs. *P* = 0.38; Supplementary Table S3). We did not examine publication bias among women with endometriosis because the number of included articles was less than 10.

## Discussion

### Findings and interpretations

Our study found that LNG-IUS may be more effective in alleviating adenomyosis-associated dysmenorrhea compared to systemic medication at 3, 6, 9, and 12 months. Also, LNG-IUS may be superior to systemic medication in reducing uterine blood loss at 3, 6, and 12 months among women with adenomyosis. However, among women with endometriosis, we observed no significant differences in the VAS score of LNG-IUS vs. systemic medication at 6 and 12 months. Most of the studies included did not report the method of randomization which indicates the low quality of these researches but the sensitivity analysis did not modify the results of our review when we excluded the studies without specific randomization method.

Dysmenorrhea includes both primary and secondary dysmenorrhea. The underlying pathology of primary dysmenorrhea is the increased local secretion of vasopressin and prostaglandins (77). Secondary dysmenorrhea is painful menses caused by pelvic pathology, most commonly endometriosis, followed by adenomyosis, infection, and myomas (2). Although the inclusion criteria for our study contained both primary and secondary dysmenorrhea, after screening abstracts and full-texts, we found no RCTs evaluating the effectiveness of LNG-IUS in primary dysmenorrhea. The probable reason may be that LNG-IUS’s usage is relatively low in primary dysmenorrhea as discussed previously (87).

According to the results, LNG-IUS might be more effective for pain relief in women with adenomyosis, which is in accordance with findings of several existing studies (9, 88). The exact mechanism of pain control in adenomyosis is still unclear, but it may be associated with the high concentration of LNG on the endometrium (9), which induces glandular atrophy and stromal decaudation, inhibiting prostaglandin synthesis (8). Prostaglandin is a substance that causes pain and uterine contraction while high levels of LNG inhibit prostaglandin synthesis.

Various potential mechanisms have been proposed for this device in women with endometriosis. Whatever the exact mechanism is, the local effect of the progestogen on the endometrium resulting in hypomenorrhoea or amenorrhoea significantly improves the pain of dysmenorrhoea and menorrhagia. A common adverse event of LNG-IUS is irregular vaginal bleeding (75). LNG-IUS groups were associated with higher risk of irregular vaginal bleeding (26.8% vs. 0) in women with endometriosis. However, there were no differences in irregular vaginal bleeding between the systemic medication and LNG-IUS groups in adenomyosis. Furthermore, there were no differences of incidence of headache, acne, or amenorrhea between the systemic medication and LNG-IUS groups. The systemic medication group was associated with a higher risk of nausea (RR:0.23, 95% CI: 0.08–0.64, *P* = 0.95) and menorrhagia (RR:0.55, 95% CI: 0.30–0.98, *P* = 0.04) in adenomyosis. We could not draw any conclusions according to available data because few studies (18, 28, 74, 75, 83) have reported on quality of life and the scales they adopted differed. More consistent studies are warranted to verify the impact of LNG-IUS on quality of life. Therefore, we suggest LNG-IUS should be offered to women with adenomyosis because it has the ability to alleviate dysmenorrhea in short time (less than 1 year). For women with endometriosis, then medication therapy was more recommended taking into account the higher risk of irregular vaginal bleeding.

### Strengths and limitations

Our study has several strengths. Firstly, to the best of our knowledge, this is the first meta-analysis to systematically compare the efficiency and safety through multiple outcomes of LNG-IUS and systemic medication among dysmenorrhea patients. Secondly, we have included women with both endometriosis and adenomyosis dysmenorrhea and conducted separate analyses. Thirdly, we included multiple control groups, comprehensively comparing the different systemic medication types. Finally, we conducted several pre-specified subgroup analyses to explore the potential source of heterogeneity.

The review also had some limitations. Firstly, most of our studies enrolled Chinese patients, which may limit the generalization of results to other ethnic groups. Majority of the studies included were published in Chinese, which limits their availability for non-Chinese speakers. Secondly, most included studies had a relatively high risk of bias, which may limit the reliability of meta-analysis results. Thirdly, high heterogenicity exists in our review. Although we have tried some subgroup analysis in terms of systemic medication type and status of surgery, we still failed to find the source of heterogenicity. Insufficient information concerning characteristics of included studies made us unable to conduct further subgroup analyses (e. g. BMI) to explore the source of heterogeneity, which also brought limitations to the reliability of our results. Forth, we did not include “endometriosis” or “adenomyosis” as search words because we think dysmenorrhea was probably mentioned as their symptoms, which may have greatly limited finding valid studies for the research focus.

Above all, there is still a need for more rigorous, well-designed and high-quality RCTs to investigate the long-term effect of LNG-IUS to confirm the current findings and to examine other vital outcomes such as number of dropouts and withdrawal of treatment.

## Conclusions

In conclusion, our systematic review demonstrated that the use of LNG-IUS was associated with a reduced severity of dysmenorrhea compared with systemic medication. Moreover, LNG-IUS was beneficial for improved control of menstrual blood loss and fewer adverse outcomes. At the same time, further well-designed RCTs are warranted to confirm the current findings and long-term effect of LNG-IUS in the treatment of dysmenorrhea.

## Data Availability

The raw data supporting the conclusions of this article will be made available by the authors, without undue reservation.
