# Supplementary material for: Levonorgestrel-releasing intrauterine system vs. systemic medication or blank control for women with dysmenorrhea: Systematic review and meta-analysis of randomized controlled trials
Source: Front Glob Womens Health. 2022 Nov 2;3:1013921. doi: 10.3389/fgwh.2022.1013921 (PMC9666369; doi:10.3389/fgwh.2022.1013921)
Supplement: Supplementary file 1 [file Table1.docx]

\

Table 1 Characteristics of included studies

| Study ID | Country/District | No. of sites | Type of patient | No. of patients | Length of follow-up (Months) | Age(Years) | Parity | Intervention | Control |
| --- | --- | --- | --- | --- | --- | --- | --- | --- | --- |
| Chen a 2018^18^ | China | 1 | Adenomyosis | 56 | 6 | 38.6 | NR | LNG-IUS | Methyltestosterone |
| Chen b 2018^32^ | China | 1 | Adenomyosis | 80 | 1.3.6 | 37.8 | NR | LNG-IUS | Mifepristone |
| Cheng 2018^59^ | China | 1 | Adenomyosis | 32 | 12 | 36.6 | 2.9 | LNG-IUS | Blank control |
| Dou 2019^63^ | China | 1 | Adenomyosis | 102 | 6 | 35.9 | 2.6 | LNG-IUS | Mifepristone |
| Feng 2018^46^ | China | 1 | Adenomyosis | 106 | 3.6 | 36.7 | NR | LNG-IUS | Gestrinone |
| Huang 2018^60^ | China | 1 | Adenomyosis | 62 | 6 | 39.3 | NR | LNG-IUS | Drospirosterone ethinyl estradiol |
| Jiang 2017^50^ | China | 1 | Adenomyosis | 100 | 6 | 36.5 | NR | LNG-IUS | Mifepristone |
| Jin 2017^20^ | China | 1 | Adenomyosis | 160 | 3 | NR | NR | LNG-IUS | Mifepristone |
| Kang 2018^12^ | China | 1 | Adenomyosis | 106 | 12 | 43.9 | NR | LNG-IUS | Gestrinone |
| Kong 2015^40^ | China | 1 | Adenomyosis | 100 | 6.12.18 | NR | NR | LNG-IUS | Mifepristone |
| Li a 2018^56^ | China | 1 | Adenomyosis | 60 | 6 | 35.1 | NR | LNG-IUS | desogestrel-E2 |
| Li b 2018^45^ | China | 1 | Adenomyosis | 80 | 1.6.12 | 39.3 | NR | LNG-IUS | Methyltestosterone |
| Li f 2017^41^ | China | 1 | Adenomyosis | 168 | 12 | 44.3 | NR | LNG-IUS | Gestrinone |
| Li g 2018^55^ | China | 1 | Adenomyosis | 84 | 1.3.6 | 36.6 | NR | LNG-IUS | Blank control |
| Li h 2016^65^ | China | 1 | Adenomyosis | 40 | 6 | 38.1 | NR | LNG-IUS | Methyltestosterone |
| Li i 2015^27^ | China | 1 | Adenomyosis | 98 | 3.6.9.12 | 39.5 | NR | LNG-IUS | Blank control |
| Li j 2015^13^ | China | 1 | Adenomyosis | 84 | 6 | 39.3 | 2.6 | LNG-IUS | Gestrinone |
| Liu a 2017^66^ | China | 1 | Adenomyosis | 70 | 12 | 37.3 | 1.1 | LNG-IUS | Mifepristone |
| Liu c 2016^31^ | China | 1 | Adenomyosis | 172 | 6 | 36.8 | NR | LNG-IUS | Methyltestosterone |
| Ma a 2016^35^ | China | 1 | Adenomyosis | 90 | 3.6 | 35.0 | NR | LNG-IUS | Gestrinone |
| Ma b 2014^67^ | China | 1 | Adenomyosis | 92 | 3 | 37.4 | NR | LNG-IUS | Mifepristone |
| Meng 2018^15^ | China | 1 | Adenomyosis | 86 | 6 | 44.0 | NR | LNG-IUS | Gestrinone |
| Re 2019^9^ | China | 1 | Adenomyosis | 62 | 6.12 | 42.8 | NR | LNG-IUS | Mifepristone |
| Ren 2017^51^ | China | 1 | Adenomyosis | 114 | 6 | NR | NR | LNG-IUS | Mifepristone |
| Shang 2014^42^ | China | 1 | Adenomyosis | 200 | 1.3.6 | NR | NR | LNG-IUS | Gestrinone |
| Shi 2016^33^ | China | 1 | Adenomyosis | 100 | 6 | 32.4 | 2.2 | LNG-IUS | Mifepristone |
| Sun a 2017^49^ | China | 1 | Adenomyosis | 82 | 6 | 34.2 | NR | LNG-IUS | Mifepristone |
| Sun b 2018^54^ | China | 1 | Adenomyosis | 66 | 3.6.9 | 35.6 | NR | LNG-IUS | Blank control |
| Tao a 2019^19^ | China | 1 | Adenomyosis | 30 | 6 | 36.8 | NR | LNG-IUS | Methyltestosterone |
| Tu 2012^62^ | China | 1 | Adenomyosis | 40 | 1.3.6 | NR | NR | LNG-IUS | Triptorelin |
| Tu 2016^29^ | China | 1 | Adenomyosis | 180 | 3 | 37.2 | NR | LNG-IUS | Mifepristone |
| Wang a 2017 | China | 1 | Adenomyosis | 60 | 3.6.12 | 40.2 | NR | LNG-IUS | Blank control |
| Wang b 2017^28^ | China | 1 | Adenomyosis | 110 | 6 | 32.4 | NR | LNG-IUS | Drospirosterone ethinylestradiol |
| Wang c 2019^8^ | China | 1 | Adenomyosis | 86 | 6 | 32.2 | NR | LNG-IUS | Gestrinone |
| Wang f 2018^69^ | China | 1 | Adenomyosis | 200 | 3 | 40.0 | 2.2 | LNG-IUS | desogestrel-E2 |
| Wang h 2014^34^ | China | 1 | Adenomyosis | 48 | 1.3.6 | 36.4 | NR | LNG-IUS | Methyltestosterone |
| Wang i 2015^30^ | China | 1 | Adenomyosis | 112 | 3.6.12 | 36.2 | NR | LNG-IUS | Mifepristone |
| Wu 2017^26^ | China | 1 | Adenomyosis | 90 | 3.6.12.24 | 38.2 | 2.1 | LNG-IUS | Gestrinone |
| Wu 2018^34^ | China | 1 | Adenomyosis | 100 | 6 | 31.6 | NR | LNG-IUS | Gestrinone |
| Yang 2016^70^ | China | 1 | Adenomyosis | 47 | 12 | 43.0 | NR | LNG-IUS | Mifepristone |
| Yao 2018^44^ | China | 1 | Adenomyosis | 60 | 6 | 34.9 | NR | LNG-IUS | Methyltestosterone |
| You 2017^68^ | China | 1 | Adenomyosis | 60 | 6 | 40.7 | NR | LNG-IUS | Mifepristone |
| Yu 2016 ^11^ | China | 1 | Adenomyosis | 85 | 6 | 39.3 | 2.7 | LNG-IUS | Gestrinone |
| Zeng 2016^52^ | China | 1 | Adenomyosis | 100 | 6 | 27.6 | NR | LNG-IUS | Gestrinone |
| Zhang a 2018^43^ | China | 1 | Adenomyosis | 40 | 6 | 36.1 | NR | LNG-IUS | Methyltestosterone |
| Zhang c 2017^25^ | China | 1 | Adenomyosis | 120 | 3.12 | 35.8 | NR | LNG-IUS | Gestrinone |
| Zhang e 2015^48^ | China | 1 | Adenomyosis | 80 | 6 | 43.5 | NR | LNG-IUS | Gestrinone |
| Zhou a 2018^10^ | China | 1 | Adenomyosis | 82 | 6 | 36.1 | NR | LNG-IUS | Methyltestosterone |
| Omar M. Shaaban1 2015^76^ | Egypt | 1 | Adenomyosis | 62 | 1.3.6 | 39.3 | NR | LNG-IUS | Drospirosterone ethinyl estradiol |
| Nelsilene Carvalho 2018^72^ | Brazil | 1 | Endometriosis | 103 | 6 | 34.1 | NR | LNG-IUS | ENG implant |
| Petta 2005^75^ | Brazil | 3 | Endometriosis | 82 | 6 | 30.0 | NR | LNG-IUS | GnRH analogue |
| Chen 2017^73^ | China, Taiwan | 1 | Endometriosis | 80 | 30 | 34.0 | NR | LNG-IUS | Blank control |
| Wu 2020^78^ | China | 1 | Adenomyosis | 251 | 18 | 36.3 | NR | LNG-IUS | GnRH analogue |
| Prasong 2012^77^ | Thailand | 1 | Endometriosis | 50 | 12 | 32.7 | NR | LNG-IUS | Expectant therapy |
| Yesim 2010^71^ | Turkey | 1 | Endometriosis | 40 | 1.3.6.12 | 37.6 | NR | LNG-IUS | GnRH analogue |
| Rui Alberto 2007^74^ | Brazil | 1 | Endometriosis | 22 | 6 | 30.9 | NR | LNG-IUS | GnRH analogue |
| Fu 2014^58^ | China | 1 | Adenomyosis | 108 | 24 | NR | NR | LNG-IUS | GnRH analogue |
| Li c 2019^57^ | China | 1 | Adenomyosis | 90 | 6 | 39.2 | NR | LNG-IUS | GnRH analogue |
| Li d 2018^39^ | China | 1 | Adenomyosis | 82 | 3 | 39.5 | NR | LNG-IUS | Mifepristone |
| Li e 2018^23^ | China | 1 | Adenomyosis | 100 | 24 | 40.4 | NR | LNG-IUS | Blank control |
| Liu b 2014^64^ | China | 1 | Endometriosis | 60 | 6.12.24 | NR | NR | LNG-IUS | Gestrinone |
| Sun c 2018^21^ | China | 1 | Adenomyosis | 88 | 6 | 41.1 | NR | LNG-IUS | Blank control |
| Tao b 2016^36^ | China | 1 | Adenomyosis | 140 | 6.12.24 | 38.8 | NR | LNG-IUS | Blank control |
| Tian 2017^24^ | China | 1 | Endometriosis | 80 | 6.12.18 | NR | NR | LNG-IUS | Gestrinone |
| Wang d 2016^22^ | China | 1 | Endometriosis | 108 | 12 | 32.7 | NR | LNG-IUS | Expectant therapy |
| Wang e 2015^38^ | China | 1 | Adenomyosis | 40 | 3.6 | 36.4 | NR | LNG-IUS | Blank control |
| Wang g 2018^14^ | China | 1 | Adenomyosis | 76 | 6.12 | 38.4 | NR | LNG-IUS | Blank control |
| Zhang b 2016^53^ | China | 1 | Adenomyosis | 106 | 1.3.6.12 | NR | NR | LNG-IUS | Blank control |
| Zhang d 2019^47^ | China | 1 | Adenomyosis | 114 | 12 | 36.3 | NR | LNG-IUS | Blank control |
| Zhao 2019^61^ | China | 1 | Adenomyosis | 60 | 12 | 35.4 | NR | LNG-IUS | GnRH analogue |
| Zhu 2019^17^ | China | 1 | Adenomyosis | 82 | 12 | 38.8 | NR | LNG-IUS | Blank control |

NR: Not reported; LNG-IUS: levonorgestrel-releasing intrauterine system; GnRh: Gonadotropin-releasing hormone

Table 2 Results of risk of bias assessment of included studies

| Author(year) | Adequate randomization sequence generation | Adequate allocation concealment | Blinding of participants | Blinding of caregivers | Blinding of outcome assessors | Free of infrequent missing outcome data | Free of selective outcome reporting | Free of other sources of bias |
| --- | --- | --- | --- | --- | --- | --- | --- | --- |
| Chen a 2018^18^ | Unclear | Unclear | Unclear | Unclear | Unclear | Unclear | Low risk | Low risk |
| Chen b 2018^32^ | Unclear | Low risk | Unclear | Unclear | Unclear | Unclear | Low risk | Low risk |
| Cheng 2018^59^ | Unclear | Unclear | Unclear | Unclear | Unclear | Unclear | Low risk | Low risk |
| Dou 2019^63^ | Low risk | Unclear | Unclear | Unclear | Unclear | Unclear | Low risk | Low risk |
| Feng 2018^46^ | Unclear | Unclear | Unclear | Unclear | Unclear | Unclear | Low risk | Low risk |
| Huang 2018^60^ | Unclear | Unclear | Unclear | Unclear | Unclear | High risk | Low risk | Low risk |
| Jiang 2017^50^ | Unclear | Unclear | Unclear | Unclear | Unclear | Unclear | Low risk | Low risk |
| Jin 2017^20^ | Unclear | Unclear | Unclear | Unclear | Unclear | Unclear | Low risk | Low risk |
| Kang 2018^12^ | Unclear | Unclear | Unclear | Unclear | Unclear | Unclear | Low risk | Low risk |
| Kong 2015^40^ | Unclear | Unclear | Unclear | Unclear | Unclear | Unclear | Low risk | Low risk |
| Li a 2018^56^ | Low risk | Unclear | Unclear | Unclear | Unclear | Unclear | Low risk | Low risk |
| Li b 2018^45^ | Low risk | Unclear | Unclear | Unclear | Unclear | Unclear | Low risk | Low risk |
| Li f 2017^41^ | Low risk | Unclear | Unclear | Unclear | Unclear | Unclear | Low risk | Low risk |
| Li g 2018^55^ | Low risk | Unclear | Unclear | Unclear | Unclear | Unclear | Low risk | Low risk |
| Li h 2016^65^ | Unclear | Unclear | Unclear | Unclear | Unclear | Unclear | Low risk | Low risk |
| Li i 2015^27^ | Unclear | Unclear | Unclear | Unclear | Unclear | Unclear | Low risk | Low risk |
| Li j 2015^13^ | Unclear | Unclear | Unclear | Unclear | Unclear | Unclear | Low risk | Low risk |
| Liu a 2017^66^ | Low risk | Unclear | Unclear | Unclear | Unclear | Unclear | Low risk | Low risk |
| Liu c 2016^31^ | Unclear | Unclear | Unclear | Unclear | Unclear | Unclear | Low risk | Low risk |
| Ma a 2016^35^ | Unclear | Unclear | Unclear | Unclear | Unclear | Unclear | Low risk | Low risk |
| Ma b 2014^67^ | Unclear | Unclear | Unclear | Unclear | Unclear | Unclear | Low risk | Low risk |
| Meng 2018^15^ | Unclear | Unclear | Unclear | Unclear | Unclear | Unclear | Low risk | Low risk |
| Re 2019^9^ | Unclear | Unclear | Unclear | Unclear | Unclear | Unclear | Low risk | Low risk |
| Ren 2017^51^ | Low risk | Unclear | Unclear | Unclear | Unclear | Unclear | Low risk | Low risk |
| Shang 2014^42^ | Unclear | Unclear | Unclear | Unclear | Unclear | Unclear | Low risk | Low risk |
| Shi 2016^33^ | Unclear | Unclear | Unclear | Unclear | Unclear | Unclear | Low risk | Low risk |
| Sun a 2017^49^ | Unclear | Unclear | Unclear | Unclear | Unclear | Unclear | Low risk | Low risk |
| Sun b 2018^54^ | Unclear | Unclear | Unclear | Unclear | Unclear | Unclear | Low risk | Low risk |
| Tao a 2019^19^ | Low risk | Unclear | Unclear | Unclear | Unclear | Unclear | Low risk | Low risk |
| Tu 2012^62^ | Unclear | Unclear | Unclear | Unclear | Unclear | Unclear | Low risk | Low risk |
| Tu 2016^29^ | Low risk | Unclear | Unclear | Unclear | Unclear | Unclear | Low risk | Low risk |
| Wang a 2017 | Unclear | Unclear | Unclear | Unclear | Unclear | Unclear | Low risk | Low risk |
| Wang b 2017^28^ | Unclear | Unclear | Unclear | Unclear | Unclear | Unclear | Low risk | Low risk |
| Wang c 2019^8^ | Low risk | Unclear | Unclear | Unclear | Unclear | Unclear | Low risk | Low risk |
| Wang f 2018^69^ | Unclear | Unclear | Unclear | Unclear | Unclear | Unclear | Low risk | Low risk |
| Wang h 2014^34^ | Unclear | Unclear | Unclear | Unclear | Unclear | Unclear | Low risk | Low risk |
| Wang i 2015^30^ | Unclear | Unclear | Unclear | Unclear | Unclear | Unclear | Low risk | Low risk |
| Wu 2017^26^ | Low risk | Unclear | Unclear | Unclear | Unclear | Unclear | Low risk | Low risk |
| Wu 2018^34^ | Low risk | Unclear | Unclear | Unclear | Unclear | Unclear | Low risk | Low risk |
| Yang 2016^70^ | Unclear | Unclear | Unclear | Unclear | Unclear | Unclear | Low risk | Low risk |
| Yao 2018^44^ | Low risk | Unclear | Unclear | Unclear | Unclear | Unclear | Low risk | Low risk |
| You 2017^68^ | Unclear | Unclear | Unclear | Unclear | Unclear | Unclear | Low risk | Low risk |
| Yu 2016 ^11^ | Low risk | Unclear | Unclear | Unclear | Unclear | Unclear | Low risk | Low risk |
| Zeng 2016^52^ | Low risk | Unclear | Unclear | Unclear | Unclear | Unclear | Low risk | Low risk |
| Zhang a 2018^43^ | Low risk | Unclear | Unclear | Unclear | Unclear | Unclear | Low risk | Low risk |
| Zhang c 2017^25^ | Unclear | Unclear | Unclear | Unclear | Unclear | Unclear | Low risk | Low risk |
| Zhang e 2015^48^ | Unclear | Unclear | Unclear | Unclear | Unclear | Unclear | Low risk | Low risk |
| Zhou a 2018^10^ | Unclear | Unclear | Unclear | Unclear | Unclear | Unclear | Low risk | Low risk |
| Omar M. Shaaban1 2015^76^ | Low risk | Low risk | Unclear | Unclear | Unclear | High risk | Low risk | Unclear |
| Nelsilene Carvalho 2018^72^ | Low risk | Low risk | High risk | High risk | High risk | High risk | Low risk | Unclear |
| Petta 2005^75^ | Low risk | Low risk | Unclear | Unclear | Unclear | Low risk | Low risk | Low risk |
| Chen 2017^73^ | Low risk | Low risk | Unclear | Unclear | Unclear | Unclear | Low risk | Low risk |
| Wu 2020^78^ | Unclear | Unclear | Unclear | Unclear | Unclear | Unclear | Low risk | Unclear |
| Prasong 2012^77^ | Low risk | Low risk | Low risk | Unclear | Unclear | High risk | Low risk | Low risk |
| Yesim 2010^71^ | Low risk | Low risk | Unclear | Unclear | Low risk | Unclear | Low risk | Low risk |
| Rui Alberto 2007^74^ | Low risk | Low risk | Unclear | Unclear | Unclear | Unclear | Low risk | Unclear |
| Fu 2014^58^ | Unclear | Unclear | Unclear | Unclear | Unclear | Unclear | Low risk | Low risk |
| Li c 2019^57^ | Unclear | Unclear | Unclear | Unclear | Unclear | Unclear | Low risk | Low risk |
| Li d 2018^39^ | Low risk | Unclear | Unclear | Unclear | Unclear | Unclear | Low risk | Low risk |
| Li e 2018^23^ | Unclear | Unclear | Unclear | Unclear | Unclear | Unclear | Low risk | Low risk |
| Liu b 2014^64^ | Unclear | Unclear | Unclear | Unclear | Unclear | Unclear | Low risk | Low risk |
| Sun c 2018^21^ | Unclear | Unclear | Unclear | Unclear | Unclear | Unclear | Low risk | Low risk |
| Tao b 2016^36^ | Unclear | Unclear | Unclear | Unclear | Unclear | High risk | Low risk | Low risk |
| Tian 2017^24^ | Unclear | Unclear | Unclear | Unclear | Unclear | Unclear | Low risk | Low risk |
| Wang d 2016^22^ | Low risk | Unclear | Unclear | Unclear | Unclear | Unclear | Low risk | Low risk |
| Wang e 2015^38^ | Unclear | Unclear | Unclear | Unclear | Unclear | Unclear | Low risk | Low risk |
| Wang g 2018^14^ | Low risk | Unclear | Unclear | Unclear | Unclear | Unclear | Low risk | Low risk |
| Zhang b 2016^53^ | Unclear | Unclear | Unclear | Unclear | Unclear | Unclear | Low risk | Low risk |
| Zhang d 2019^47^ | Low risk | Unclear | Unclear | Unclear | Unclear | Unclear | Low risk | Low risk |
| Zhao 2019^61^ | Low risk | Unclear | Unclear | Unclear | Unclear | Unclear | Low risk | Low risk |
| Zhu 2019^17^ | Low risk | Unclear | Unclear | Unclear | Unclear | Unclear | Low risk | Low risk |

Table 3 Sensitivity analyses of included studies based on VAS score at 6 months

|  | **Number of studies included** | **SMD (95%CI)** | **P value** |
| --- | --- | --- | --- |
| **Adenomyosis women** |  |  |  |
| Primary pooling result | 35 | -1.25(-1.58, -0.92) | <0.0001 |
| Pooling result of studies with low risk of bias from randomization | 11 | -1.24(-1.64, -0.84) | <0.001 |
| Pooling result of studies’ sample size less than 50 | 12 | -0.93 (-1.44, -0.43) | <0.00001 |
| **Endometriosis women** |  |  |  |
| Primary pooling result | 6 | -0.27(-0.97, 0.43) | 0.45 |
| Pooling result of studies with low risk of bias from randomization | 4 | -0.38(-1.22, 0.47) | 0.38 |

Table 4 Results of subgroup analysis based on VAS score at 6 months

|  | **No. of studies** | **SMD (95%CI)** | **P value of interaction test** |
| --- | --- | --- | --- |
| Subgroup by drug type | | | |
| Mifepristone | 8 | -1.7 (-2.51, -0.89) | <0.001 |
| Gestrinone | 8 | --1.29 (-1.62, -0.96) |  |
| Drospirosterone ethinylestradiol | 3 | -1.29 (-3.11, 0.52) |  |
| desogestrel-E2 | 1 | -0.08 (-0.59, 0.43) |  |
| Triptorelin | 1 | 2.17 (1.37, 2.96) |  |
| Methyltestosterone | 3 | -1.82 (-2.11, 1.52) |  |
| Blank control | 3 | -1.07(-1.66, -0.48) |  |
| Status of surgery in adenomyosis women | | | |
| Nooperation |  | -1.25 (-1.6, -0.9) | 0.91 |
| Postoperation |  | -1.30(-2.18, -0.42) |  |
| Status of surgery in endometriosis women | | | |
| Nooperation |  | 0.00 (-0.3, 0.3) | 0.42 |
| Postoperation |  | -0.69(-2.36, 0.98) |  |

Table 5 Results of publication bias (Egger’s test)

| **Outcomes** | **No. of studies** | **P** |
| --- | --- | --- |
| VAS score at 3 months of adenomyosis women | 11 | 0.17 |
| VAS score at 6 months of adenomyosis women | 27 | 0.65 |
| VAS score at 12 months of adenomyosis women | 11 | 0.21 |
| PBAC score at 6 months of adenomyosis women | 10 | 0.09 |
| Menstrual blood loss at 6 months of adenomyosis women | 15 | 0.73 |
| Acne of adenomyosis women | 11 | 0.36 |
| Irregular vaginal bleeding of adenomyosis women | 12 | 0.27 |


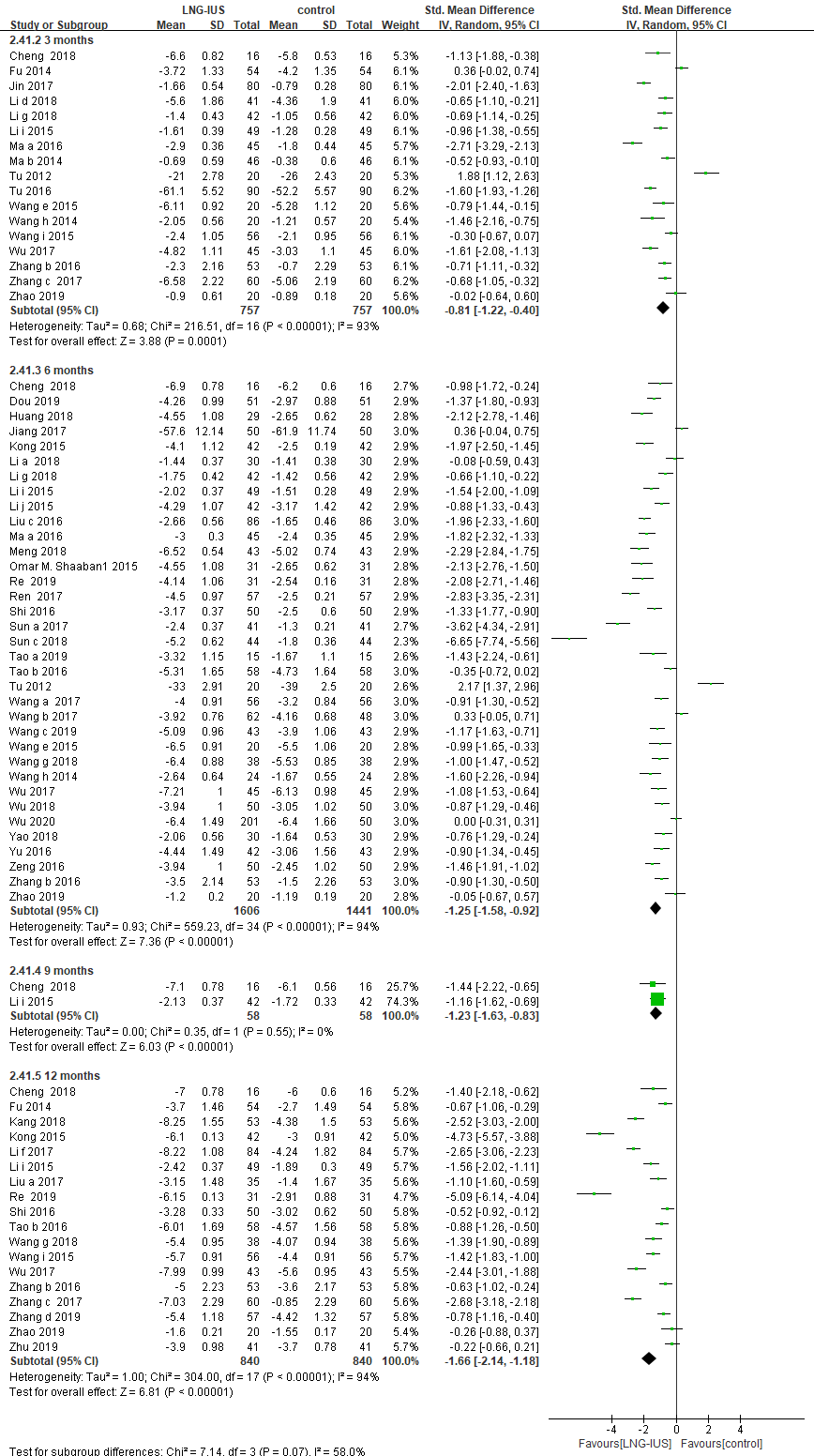
Figure 2 VAS score among adenomyosis patients receiving treatment of LNG-IUS versus medication in randomized controlled study


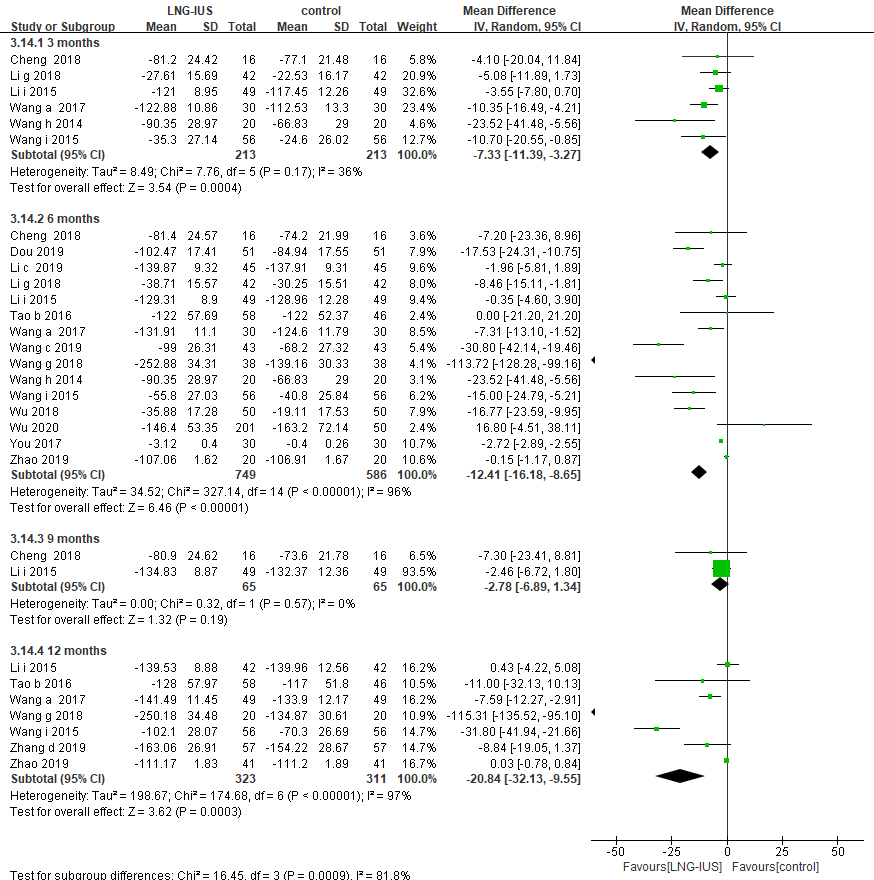
Figure 3 PBAC among adenomyosis patients receiving treatment of LNG-IUS versus medication in randomized controlled study


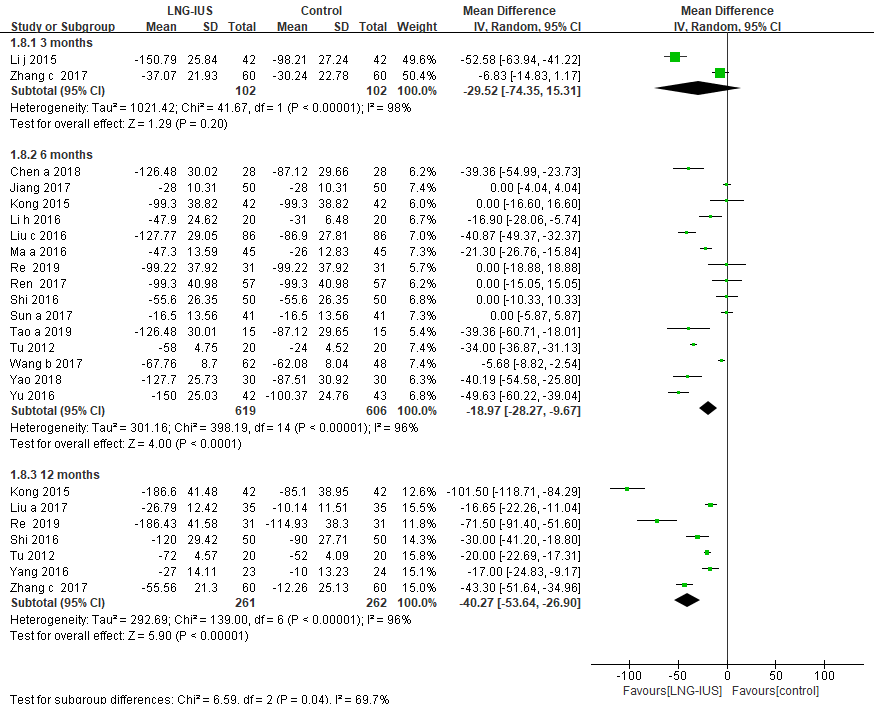


Figure 4 Menstrual blood loss among adenomyosis patients receiving treatment of LNG-IUS versus medication in randomized controlled study


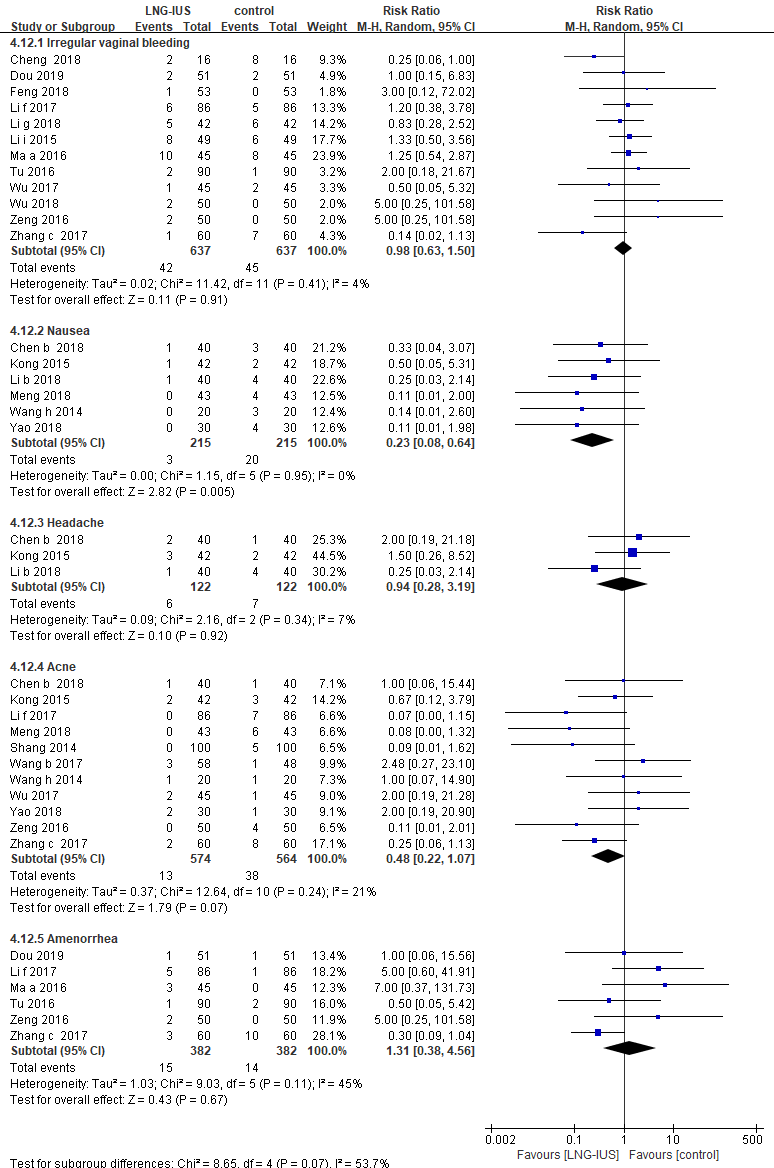


Figure 5 Adverse events among adenomyosis patients receiving treatment of LNG-IUS versus medication in randomized controlled study


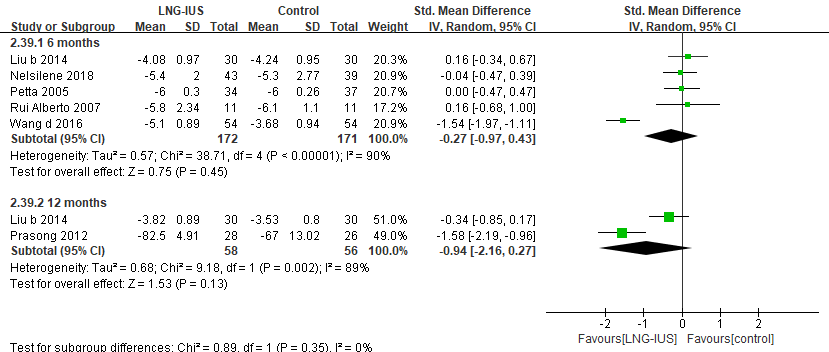


Figure 6 VAS score among endometriosis patients receiving treatment of LNG-IUS versus medication in randomized controlled study


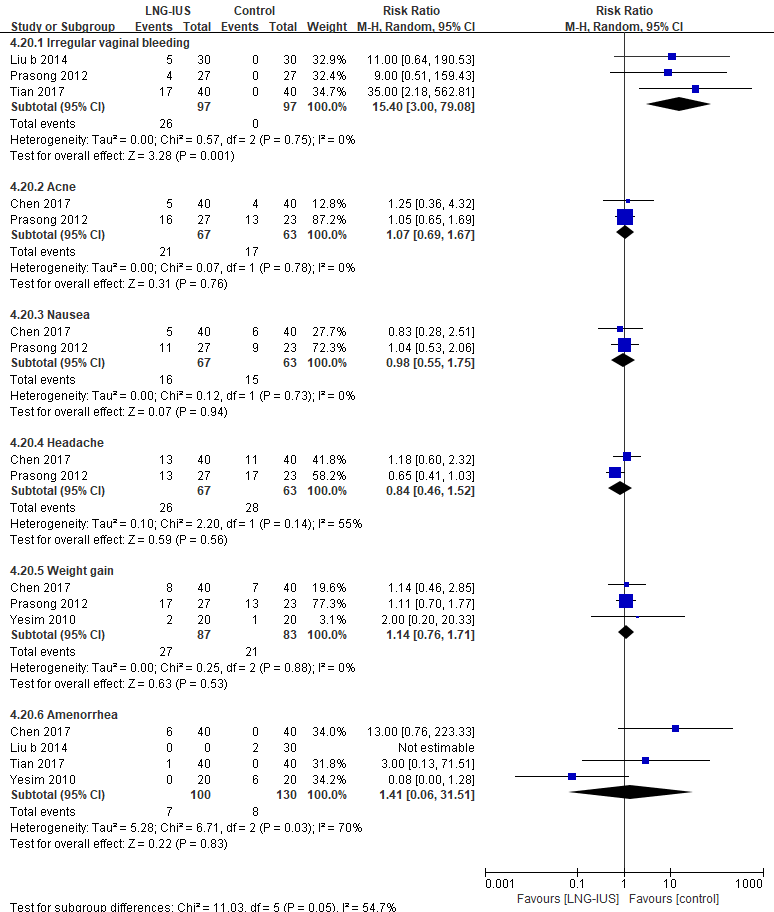


Figure 7 Adverse events among endometriosis patients receiving LNG-IUS versus medication

**Search strategy**

1. **Pubmed**

1. mirena

2. levonorgestrel releasing intrauterine system

3. LNG-IUS

4. levonorgestrel intrauterine system

5. 1 or 2 or 3 or 4

6. dysmenorrhea

7. painful menses

8. menstrual pain

9. dyspareunia

10. chronic pelvic pain

11. 6 or 7 or 8 or 9 or 1o

12. 5 and 11

1. **EMBASE**

1. mirena.mp. or mirena/

2. levonorgestrel releasing intrauterine system/ or levonorgestrel releasing intrauterine system.mp.

3. LNG-IUS.mp. or LNG-IUS/

4. levonorgestrel intrauterine system.mp. or levonorgestrel intrauterine system/

5. 1 or 2 or 3 or 4

6. dysmenorrhea mp. or dysmenorrhea/

7. painful menses mp. or painful menses/

8. dyspareunia mp. or dyspareunia/

9. menstrual pain mp. or menstrual pain/

10. chronic pelvic pain mp. or chronic pelvic pain/

11. 6 or 7 or 8 or 9 or 10

12. 5 and 11

1. **CNKI**

(SU='Mirena'+ 'LNG-IUS') AND (SU='dysmenorrhea'+ 'chronic pelvic pain')

1. **Wanfang Data**

(SU='Mirena'+ 'LNG-IUS') AND (SU='dysmenorrhea'+ 'chronic pelvic pain')
